# Crime Exposure, Dementia Risks, and Racial and Ethnic Disparities Among Middle-Aged and Older Americans

**DOI:** 10.1093/geroni/igaf122.167

**Published:** 2025-12-31

**Authors:** Junjie He, Zhuoer Lin, Yan Zhang, Yang Wang, Xi Chen

**Affiliations:** Yale University, State College, Pennsylvania, United States; University of Illinois Chicago, Chicago, Illinois, United States; Texas State University, Greenville, North Carolina, United States; University of Wisconsin–Madison, Madison, Wisconsin, United States; Yale University, New Haven, Connecticut, United States

## Abstract

Exposure to crime, a significant social stressor, may profoundly affect cognitive functioning, particularly among racial and ethnic minorities, yet its long-term relationship with dementia risk remains underexplored. In this study, we examined the association between crime exposure and the incidence of cognitive impairment and dementia, with a focus on racial and ethnic disparities. We analyzed data from the Health and Retirement Study, linking a nationally representative sample of Americans aged 50 + (N = 34,625, or 177,238 person-waves) with local crime rates from the Uniform Crime Reporting system (1995-2016). Crime exposure was measured at the county-year level, accounting for both current and prior year exposures to assess both immediate and cumulative effects. Cognitive outcomes were determined using a validated algorithm based on cognitive test scores and proxy assessments. Using time-varying, weighted Cox proportional hazards models, we adjusted for individual and census track-level factors and accounted for state and racial and ethnic stratifications. Our findings revealed that higher local crime rates were associated with increased risks of cognitive impairment (HR 1.29 per IQR; 95% CI, 1.21-1.38) and dementia (HR 1.23 per IQR; 95% CI, 1.14-1.32), with property crimes showing stronger associations than violent crimes. Non-Hispanic Black and Hispanic individuals experienced significantly greater associations between crime exposure and cognitive outcomes compared to non-Hispanic White individuals. These results underscore the need for targeted interventions to address structural racial and ethnic inequities in neighborhood crime exposures and associated cognitive outcomes, particularly among racial and ethnic minorities.

